# Current-Induced Field-Free Switching of Co/Pt Multilayer via Modulation of Interlayer Exchange Coupling and Magnetic Anisotropy

**DOI:** 10.3390/ma17215214

**Published:** 2024-10-25

**Authors:** Byungro Kim, Dongpyo Seo, Seungha Yoon, Songhee Han, Taeheon Kim, Beongki Cho

**Affiliations:** 1 School of Materials Science and Engineering, Gwangju Institute of Science and Technology, 123 Cheomdangwagi-ro, Buk-gu, Gwangju 61005, Republic of Korea; kbr7874@gist.ac.kr (B.K.);; 2 Energy and Nano R&D Group, Korea Institute of Industrial Technology, Cheomdangwagi-ro 208-gil, Buk-gu, Gwagju 61012, Republic of Korea; yoonsh@kitech.re.kr; 3 Division of Navigation Science, Mokpo National Maritime University, Mokpo 58628, Republic of Korea; 4 Electro-Medical Equipment Research Division, Applied Electromagnetic Wave Research Center, Korea Electrotechnology Research Institute, Ansan 15588, Republic of Korea; thkim23@keri.re.kr

**Keywords:** spintronics, spin Hall effect, spin-transfer torque, epitaxial film, magnetic reversal

## Abstract

Current-induced field-free magnetic switching using spin–orbit torque has been an important topic for decades due to both academic and industrial interest. Most research has focused on introducing symmetry breakers, such as geometrical and compositional variation, pinned layers, and symmetry-broken crystal structures, which add complexity to the magnetic structure and fabrication process. We designed a relatively simple magnetic structure, composed of a [Co/Pt] multilayer and a Co layer with perpendicular and in-plane magnetic anisotropy, respectively, with a Cu layer between them. Current-induced deterministic magnetic switching was observed in this magnetic system. The system is advantageous due to its easy control of the parameters to achieve the optimal condition for magnetic switching. The balance between magnetic anisotropic strength and interlayer coupling strength is found to provide the optimal condition. This simple design and easy adjustability open various possibilities for magnetic structures in spin-based electronics applications using spin–orbit torque.

## 1. Introduction

Field-free magnetic switching, using spin–orbit torque (SOT), has emerged as a promising technique in spintronics, particularly for applications in magnetic random-access memory (MRAM) [1,2,3], spin logic devices [4,5], and spin oscillators [6,7]. The concept of SOT involves the use of spin currents generated by the spin Hall effect or Rashba–Edelstein effect to manipulate the magnetization of a ferromagnetic layer. This method offers significant advantages over traditional spin-valve-type MRAMs, which typically require an external magnetic field for deterministic switching, and over spin-transfer torque (STT) mechanisms [8].

The exploration of SOT began in the early 2000s, with initial studies focusing on heavy metals like platinum (Pt) and tantalum (Ta) due to their strong spin–orbit coupling [9,10,11]. Over the years, researchers have demonstrated the potential of SOT in achieving efficient magnetization switching. A significant milestone was the realization of field-free switching by engineering the symmetry of the magnetic heterostructures, such as using the canted deposition of materials like molybdenum (Mo) and tungsten (W) [12,13,14].

One of the primary purpose of using the SOT effect is to induce magnetic switching without an external magnetic field, which simplifies device architecture and reduces power consumption [15,16,17]. Additionally, SOT offers faster switching speeds and higher endurance compared to STT, making it suitable for high-performance memory applications [8]. The ability to achieve deterministic switching through structural engineering, rather than relying solely on material properties, opens new avenues for device optimization.

Despite its advantages, SOT-based switching faces several challenges. The efficiency of spin-current generation and transfer is highly dependent on the materials and their interfaces, which can complicate the fabrication process. Moreover, symmetry breaking is necessary to achieve uniform and reliable switching across magnetic layers with perpendicular magnetic anisotropy. Researchers have explored diverse approaches, such as synthetic antiferromagnetic systems [18], magnetic tunnel junctions with 2D materials [19], in-plane exchange bias [20], compositional variations in magnetic layers [21], etc. The need for precise control over the growth and deposition of materials adds complexity and costs to manufacturing SOT electronic devices.

The Co/Pt multilayer system exhibits several interesting magnetic properties, making it a subject of extensive research in the field of spintronics and magnetic storage technologies. Co/Pt multilayers are known for their strong perpendicular magnetic anisotropy, which means the magnetic moments prefer to align perpendicular to the plane of the layers [22]. This property is crucial for high-density magnetic storage applications. The magnetization and coercivity of Co/Pt multilayers can be tuned by varying the thickness of the Co and Pt layers. Thicker Co layers tend to increase the in-plane magnetic anisotropy, while thinner layers enhance the perpendicular anisotropy. The coercivity, which is the resistance to changes in magnetization direction, can also be significantly high, especially when the layers are grown at elevated temperatures [23]. Coupling between Co and Pt layers can lead to complex magnetic behaviors. For instance, the presence of Pt can mediate ferromagnetic coupling between Co layers, and its coupling strength and direction of spin alignment shows oscillatory change, depending on nonmagnetic spacer thickness. It is described by the Ruderman–Kittel–Kasuya–Yosida (RKKY) theory [24,25,26,27]. These properties make Co/Pt multilayers a versatile and valuable system for both fundamental research and practical applications in magnetic storage and spintronic devices. In this work, we demonstrate field-free SOT magnetic switching in a [Co/Pt]/Cu/Co system. By optimizing the thicknesses of the Cu and Pt layers to adjust the interlayer exchange coupling and magnetic anisotropy strength, respectively, we achieved the optimal conditions for reliable switching.

## 2. Experimental Details

**Figure 1:**
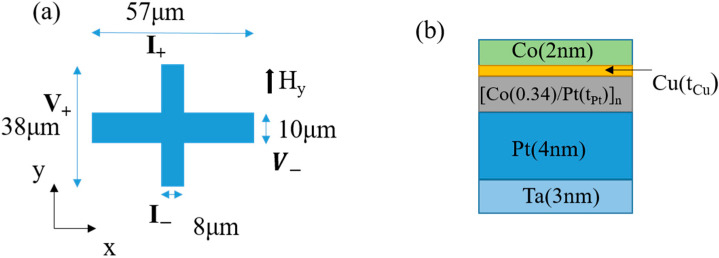
Schematic sample layout of (**a**) the top view of the Hall bar and (**b**) the layer sequence in the cross section of the Hall bar.

Figure 1a,b illustrate the schematic structure for Hall measurement and the layer stack sequence, respectively. The Hall bar consists of two sections: one with dimensions of 10 μm×57 μm for the Hall voltage, and another with dimensions of 8 μm×38 μm for the current flow measurement.

The magnetic layer stack sequence is Ta(3)/Pt(4)/${\left[Co(0.34)/Pt(t_{Pt})\right]}_{n}$/Cu($t_{Cu}$)/Co(2), where the numbers in parentheses represent the thickness of each layer in nanometers. Here, $t_{Pt}$ and $t_{Cu}$ denote the variable thicknesses of the Pt and Cu layers, respectively, and n is the number of Co/Pt layers. The nominal thickness of layers was determined by sputtering time with a controlled target growth rate. The Ta layer serves as a buffer at the bottom. The thick Pt layer above the Ta buffer acts as a heavy metal to generate the spin Hall effect. The Cu layer, instead of Pt (or Ru), was used as an interface spacer because it reduced the out-of-plane anisotropy of a single Co(2) layer and had almost no spin Hall angle, meaning it had a negligible SOT effect on the Co layer [28,29].

A magnetron sputtering system with an initial base pressure of $1\times 10^{-8}\;torr$ was used to fabricate samples without breaking the vacuum throughout the entire deposition process. To prevent structural asymmetry due to possible oblique deposition, we rotated the sample holder during the deposition. In addition, the deposition process was carefully controlled to minimize potential interlayer Dzyaloshinskii–Moriya interaction. For Hall effect measurement, we used a Keithly 6221 (Cleveland, OH, USA) current source and Keithly 2182A (Cleveland, OH, USA) nanovoltmeter. Magnetic measurements were conducted using the Lakeshore VSM model 7400 series (Westerville, OH, USA).

## 3. Results and Discussion

**Figure 2:**
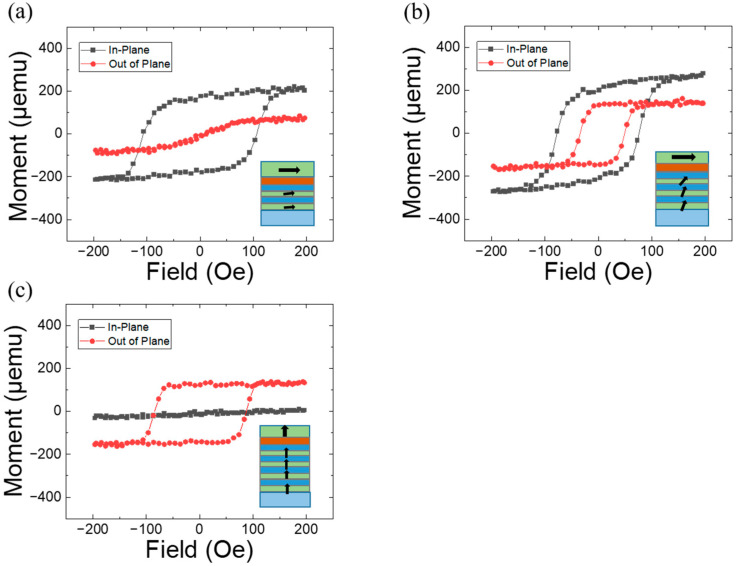
Magnetization as a function of the applied field for a magnetic system of $Ta(3)/Pt(4)/{\left[Co(0.34)/Pt(t_{Pt})\right]}_{n}/Cu(t_{Cu})/Co(2)$: (**a**) $t_{Pt}=0.8$, $t_{Cu}=0.6$, and n=2; (**b**) $t_{Pt}=0.8$, $t_{Cu}=0.6$, and n=3; and (**c**) $t_{Pt}=0.8$, $t_{Cu}=0.6$, and n=4. The inset shows the probable schematic magnetic configuration in each condition.

Figure 2 shows the magnetic hysteresis loops of magnetic stacks with $t_{Pt}=0.8$, $t_{Cu}=0.6$, and n=2, 3, and 4. The multilayers with n=2 and 4 exhibit only in-plane magnetic anisotropy (IMA) (Figure 2a) and out-of-plane magnetic anisotropy (OMA) (Figure 2c), respectively. In contrast, the multilayer with n=3 displays both IMA and OMA (Figure 2b). Since the ${\left[Co/Pt\right]}_{n}$ multilayer and the Co layer inherently possess OMA and IMA, respectively, the data in Figure 2 suggest a strong interlayer coupling between them, specifically through an exchange interaction via the Cu space layer. For ${\left[Co/Pt\right]}_{n=2}$, this interlayer coupling causes the perpendicular magnetic moments to rotate in-plane. Conversely, for ${\left[Co/Pt\right]}_{n=4}$, the coupling induces the in-plane magnetic moments of the Co layer to rotate perpendicular to the plane. The effective magnetic anisotropy energy values for n=2 and n=4 were estimated to be $1.17\times 10^{5}\;erg/{cm}^{3}$ and $2.44\times 10^{5}\;erg/{cm}^{3}$, respectively. For ${\left[Co/Pt\right]}_{n=3}$, the IMA in the Co layer and the OMA in the Co/Pt layer compete to maintain their respective magnetic anisotropies. Given the strong interlayer coupling, it is inferred that the magnetic anisotropy strength on both sides is significantly reduced. The coercive field for IMA in Figure 2b (≈85 Oe) is smaller than that in Figure 2a (≈100 Oe), and the coercive field for OMA in Figure 2b (≈35 Oe) is smaller than that in Figure 2c (≈90 Oe). The variations in the coercive field are consistent with the scenario of reduced magnetic anisotropy strength.

**Figure 3:**
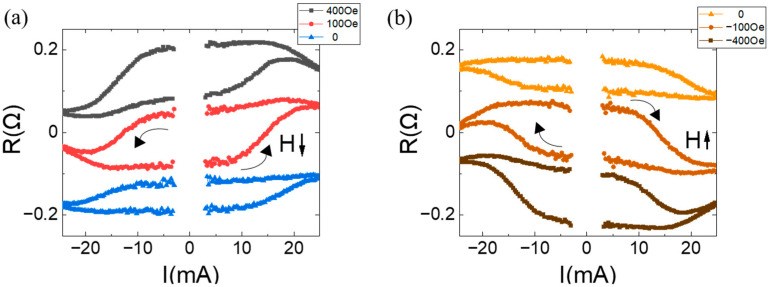
Anomalous Hall resistance as a function of the applied current for a magnetic system of $Ta(3)/Pt(4)/{\left[Co(0.34)/Pt(t_{Pt})\right]}_{n}/Cu(t_{Cu})/Co(2)$ with $t_{Pt}=0.8$, $t_{Cu}=0.6$, and n=3, under the specified external fields: (**a**) field along + *y*-axis and (**b**) field along − *y*-axis. The data are shifted off for comparison.

Figure 3a shows the Hall resistance $\left(R_{H}\right)$ as a function of current for the sample with $t_{Pt}=0.8$, $t_{Cu}=0.6$, and n=3 under various fields of 0, 100, and 400 Oe. The current pulse with a duration of 1 ms is applied along the + *y*-axis to the Hall bar structure depicted in Figure 1a. The Hall resistance difference is defined as $∆R(H)\equiv \left|R_{+}-R_{-}\right|$, where $R_{H+}$ and $R_{H-}$ are the resistances when current flows along the positive and negative *y*-axis, respectively. Because the Hall resistance is due to the anomalous Hall effect induced by the magnetization in the Co/Pt multilayer, the difference indicates magnetic moment polarity switching induced by the combination of applied current and field. For H=0 Oe, ∆R(H=0) is observed to be 0.063 Ω, indicating that the magnetic polarity of the Co/Ft multilayer is deterministically switched solely by the applied current. When H=100 Oe, ∆R(H=100) increases to 0.119 Ω, suggesting that the field enhances the magnetic switching. No further enhancement was observed with an increase in the applied field, i.e., ∆R(H=100)≈∆R(H=400). The switching ratio is defined as ∆R(H=0)/∆R(H=400) to be ≈0.53. The effective internal field was estimated to be ≈40 Oe.

The same measurements were performed for the samples with $t_{Pt}=0.8$, $t_{Cu}=0.6$ and n=2 and 4. For n=2, no magnetic polarity change was observed. For n=4, a magnetic polarity change was observed only when a magnetic field was applied, indicating no zero-field switching. In this case, the externally applied field acts as a symmetry breaker for current-induced magnetic switching. Therefore, the magnetic structure with $t_{Pt}=0.8$, $t_{Cu}=0.6$, and n=3 has appropriate interlayer coupling between the Co/Pt multilayer and the Co layer, providing an effective internal field as a symmetry breaker, making current-induced magnetic switching possible.

Figure 3b shows the Hall resistance with the field along the − *y*-axis. Compared to the $R_{H}$ data in Figure 3a, there is almost no change in $R_{H}$ behaviors with the fields of 0, −100, and −400 Oe. This indicates that the sample in this study is free from field-dependent directional symmetries, such as geometrical gradient, compositional inhomogeneity, and the interface Dzyaloshinskii–Moriya interaction (DMI) effect, for current-induced magnetic switching, other than the internal field due to interlayer coupling. The internal field was found to be biaxial along the *y*-axis.

**Figure 4:**
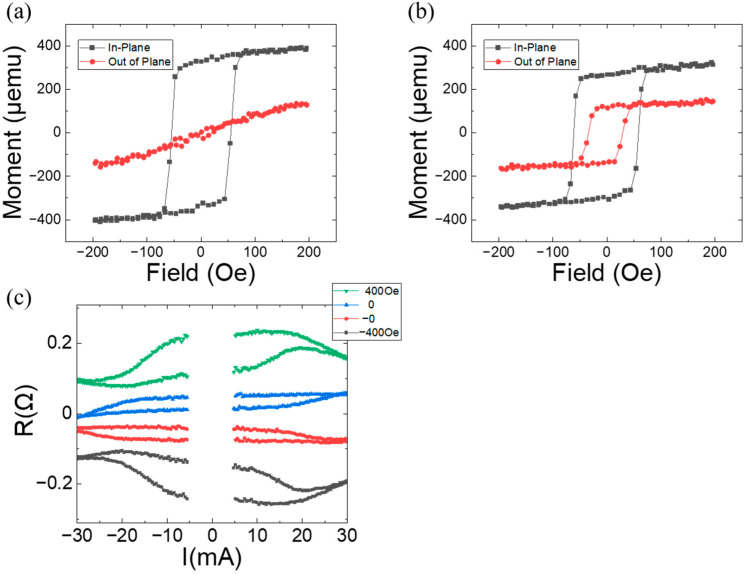
Magnetization as a function of the applied field for a magnetic system of $Ta(3)/Pt(4)/{\left[Co(0.34)/Pt(t_{Pt})\right]}_{n}/Cu(t_{Cu})/Co(2)$: (**a**) $t_{Pt}=0.8$, $t_{Cu}=0.3$, and n=3 and (**b**) $t_{Pt}=0.8$, $t_{Cu}=0.9$, and n=3. (**c**) Anomalous Hall resistance as a function of the applied current with specified external fields parallel to the current direction (the *y*-axis) for the sample of (**b**). The data are shifted off for comparison.

To investigate the effects of Cu space layer thickness on magnetic polarity, samples with $t_{Pt}=0.8$, n=3, and $t_{Cu}=0.3$ and 0.9 were fabricated. Their magnetizations are plotted as a function of the fields in Figure 4a,b. For $t_{Cu}=0.3$, the sample lost its OMA characteristics in the Co/Pt multilayer, and no magnetic polarity switching was observed, as expected. For $t_{Cu}=0.9$, both IMA in the Co layer and OMA in the Co/Pt multilayer were maintained. Hall bar measurements were performed and are plotted in Figure 4c. Field-free switching was observed with a value of $∆R\left(H=0\right)=0.034$, which is much smaller than that in Figure 3. The ∆R values reached their maximum at H=+400 and −400 Oe. The switching ratio is ≈0.3. It can be noted that a decrease in Cu layer thickness enhances interlayer coupling, destroying OMA in the Co/Pt multilayer, and that an increase weakens interlayer coupling, reducing the effective internal field (≈15 Oe) for current-induced switching, and maintaining the coercive field almost unchanged.

**Figure 5:**
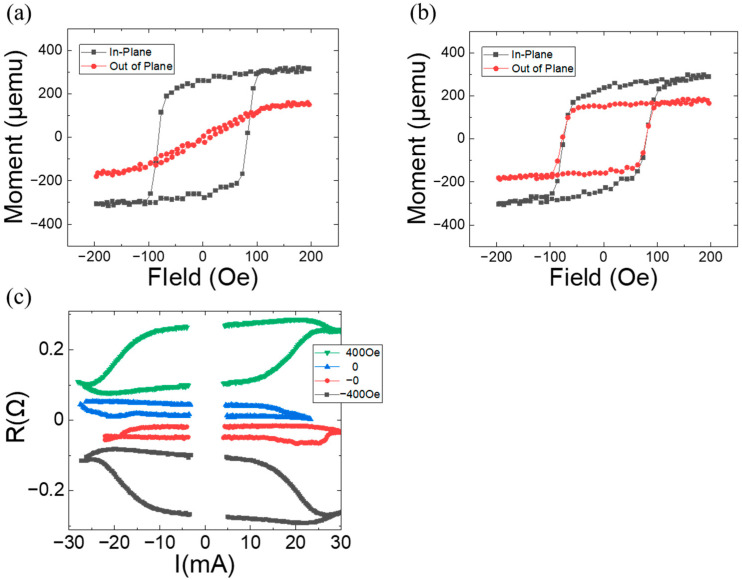
Magnetization as a function of the applied field for a magnetic system of $Ta(3)/Pt(4)/{\left[Co(0.34)/Pt(t_{Pt})\right]}_{n}/Cu(t_{Cu})/Co(2)$: (**a**) $t_{Pt}=0.6$, $t_{Cu}=0.6$, and n=3, and (**b**) $t_{Pt}=1.0$, $t_{Cu}=0.6$, and n=3. (**c**) Anomalous Hall resistance as a function of the applied current with specified external fields parallel to current direction (the *y*-axis) for the sample of (**b**). The data are shifted off for comparison.

Given the significant dependence of OMA characteristics on Pt thickness in Co/Pt multilayers, samples with $t_{Cu}=0.6$, n=3, and $t_{Pt}=0.6$ and 1.0 were fabricated. Their magnetization is plotted as a function of the field in Figure 5a,b. For $t_{Pt}=0.6$, the sample loses its OMA characteristics in the Co/Pt multilayer. Assuming that interlayer coupling through the Cu space layer ($t_{Cu}=0.6$) is the same as in Figure 2, the loss of OMA is likely due to the reduction in OMA strength with a smaller Pt layer thickness. The reduction is consistent with the results in reference [22]. For $t_{Pt}=1.0$, both IMA in the Co layer and OMA in the Co/Pt multilayer are maintained. The coercive field in OMA magnetization significantly increases compared to that in Figure 2b, indicating enhanced magnetic anisotropy strength. Hall bar measurements were performed and are plotted in Figure 5c. Field-free switching is observed with a value of $∆R\left(H=0\right)=0.034$, which is much smaller than that in Figure 3 and comparable to that in Figure 4. ∆R values reach their maximum at H=+400 and −400 Oe. The switching ratio is found to be ≈0.19. The condition of the effective internal field (≈25 Oe) from $t_{Cu}=0.6$ and OMA strength from $t_{Pt}=1.0$ is not optimal for yielding magnetic switching as effectively as in Figure 3b.

**Figure 6:**
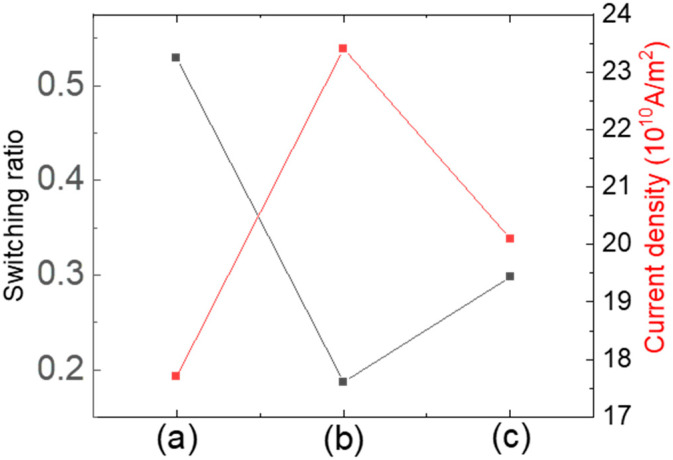
Magnetic switching ratio of zero-field switching (black symbols, left vertical axis) to the magnetic full switching and critical current density (red symbols, right vertical axis) for the samples: (**a**) $t_{Pt}=0.8$, $t_{Cu}=0.6$, and n=3; (**b**) $t_{Pt}=1.0$, $t_{Cu}=0.6$, and n=3; and (**c**) $t_{Pt}=0.8$, $t_{Cu}=0.9$, and n=3.

The zero-field switching ratios in Figure 3, Figure 4 and Figure 5, along with the critical current density for switching, are summarized in Figure 6 for comparison. The critical current density was calculated assuming a uniform distribution of current across the sample.

The sample with $t_{Pt}=0.8$, $t_{Cu}=0.6$; and n=3 shows the highest switching ratio (≈0.53) and the lowest critical current density ($17.7\times 10^{10}\;A/m^{2}$). In contrast, the sample with $t_{Pt}=1.0$, $t_{Cu}=0.6$, and n=3 exhibits the lowest switching ratio (≈0.19) and the highest critical current density ($23.4\times 10^{10}\;A/m^{2}$). The sample with $t_{Pt}=0.8$, $t_{Cu}=0.9$, and n=3 has a switching ratio of ≈0.3 and a critical current density of $20.1\times 10^{10}\;A/m^{2}$. The lowest critical current density is comparable to the value (≈$27.41\times 10^{10}\;A/m^{2})$ reported in reference [30], which used the SOT effect for switching. It has also been reported that a magnetic system utilizing topological insulators exhibits a smaller value (≈$1.5\times 10^{9}\;A/m^{2})$ [31]. Any degradation in switching behavior was not observed after repeated measurements with the applied current, indicating stable switching at room temperature. Additionally, deviating from the optimal values for both anisotropy and interlayer coupling reduces the effectiveness of magnetic switching, with magnetic anisotropy having a more detrimental effect than interlayer coupling.

## 4. Conclusions

We report uniform and reliable current-induced field-free switching in a magnetic multilayer system composed of $Ta(3)/Pt(4)/{\left[Co(0.34)/Pt(t_{Pt})\right]}_{n}/Cu(t_{Cu})/Co(2)$. The magnetic anisotropy strength of the Co/Pt layer can be controlled by varying the number of repetitions (n) and the pt layer thickness ${(t}_{Pt})$. Additionally, the interlayer coupling strength between the Co/Pt and Co layers can be adjusted by changing the Cu layer thickness $\left(t_{Cu}\right)$. Field-free reliable magnetic switching was achieved with the parameter values $t_{Pt}=0.8$, $t_{Cu}=0.6$, and n=3, which provided the optimal conditions for magnetic switching by balancing magnetic anisotropy and interlayer coupling strength. Although the other samples with $t_{Pt}=1.0$, $t_{Cu}=0.6$, and n=3, and $t_{Pt}=0.8$, $t_{Cu}=0.9$, and n=3, exhibited field-free switching, it was not as effective as the optimal condition.

Using a Co/Pt multilayer with perpendicular magnetic anisotropy and a Co layer for interlayer coupling via a Cu spacer layer exhibited interesting features for field-free magnetic switching: a simple structural design, uniform deposition in the fabrication process, biaxial symmetry breaking, and optimization by adjusting appropriate parameters. Our results open possibilities for various designs in SOT-based electronics.

## Data Availability

Data are contained within the article.
